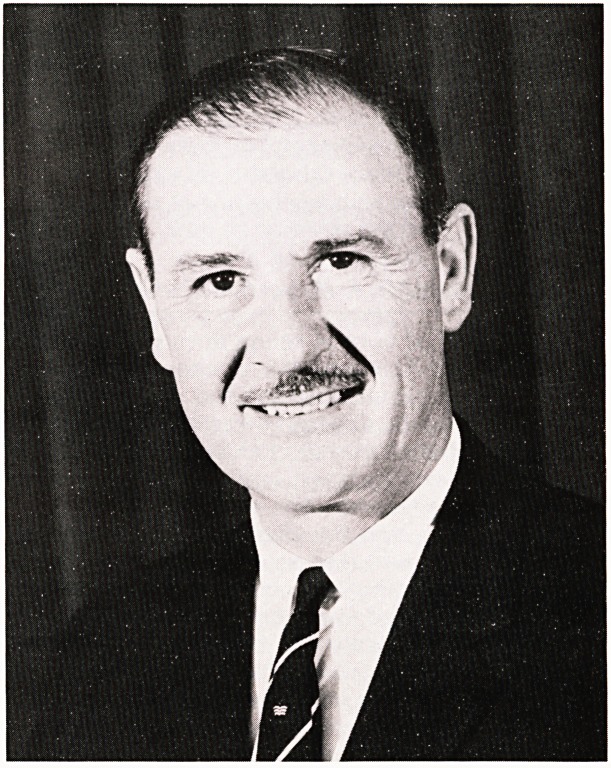# Mr. Douglas Mearns Milne

**Published:** 1982

**Authors:** 


### Obituary

### Mr. Douglas Mearn Milne, K.St.J., M.B., Ch.B., F.R.C.S.(Ed.& Eng.)

On 14th January 1982 Douglas Mearns Milne, Consultant Thoracic Surgeon to Frenchay Hospital and the South Western Regional Health Authority, died at the age of 65 years after a very short illness. He had in fact just reached official retirement age, but was serving, by invitation, an extra year at Frenchay Hospital.

Douglas Milne was born on 16th August 1916 in Montrose, where his father was a general practitioner. He was educated at Montrose Academy and graduated in medicine at Aberdeen University in 1940. After House appointments, he joined the RAMC in 1941 and spent the rest of the war on Active service in the Middle East. It was in 1945, while serving in No.4 General Hospital, Quassassin, Egypt, that he met and married his wife, Margaret, who was a nursing sister in the Queen Alexandra's Imperial Military Nursing Service. Soon after demobilisation in 1947 he obtained the F.R.C.S.(Ed.) and subsequently trained in Thoracic Surgery, first in Aberdeen and then at Frenchay Hospital under Ronald Belsey. In 1952 he was appointed Consultant Surgeon to Frenchay Hospital and to the Taunton Area of the South West Region. In 1976 he was awarded the Honorary Fellowship of the Royal College of Surgeons of England.

Although technically a superb general thoracic surgeon whose advice and operative skills were in great demand, he will be particularly remembered as a radical and courageous oesophageal surgeon. No matter how old or frail the patient, he regarded malignant dysphagia as a personal challenge, and his experience with this condition was almost without parallel. He provided worthwhile palliation for numerous patients and at low risk, an achievement of which he was justly proud. He never considered himself to be an academic surgeon; although he reported his wide experience of surgery of benign and malignant strictures of the oesophagus and of the management of chest-wall deformities, he was more comfortable by the bedside or in the operating theatre.

Douglas had many interests outside medicine, of which the greatest undoubtedly was association football. He was a director of Bristol Rovers from 1961 and their Chairman from 1968 to 1978. He was an excellent golfer, a past Captain of Long Ashton Golf Club and a keen fisherman, when time allowed. He was a competent violinist and a fluent composer of light verse, who enlivened many festive occasions with his rhyming couplets. He was a senior member of the St. John Ambulance and had been created a Knight of the Order of St. John shortly before his death.

Douglas had a powerful and outgoing personality that endeared him to his colleagues, stimulated his juniors and comforted numerous patients. He was blessed with a very happy family life with his wife Margaret, who nursed him skilfully and devotedly during his final illness and who survives him together with their son and two daughters.

G.K., A.T.M.R.

## Figures and Tables

**Figure f1:**